# Hyperspectral venous image quality assessment for optimum illumination range selection based on skin tone characteristics

**DOI:** 10.1186/1475-925X-13-109

**Published:** 2014-08-03

**Authors:** Aamir Shahzad, Mohamad Naufal Saad, Nicolas Walter, Aamir Saeed Malik, Fabrice Meriaudeau

**Affiliations:** 1 Centre for Intelligent Signal and Imaging Research (CISIR), Department of Electrical and Electronics Engineering, Universiti Teknologi PETRONAS, 31750 Tronoh Perak, Malaysia; 2 Le2i Laboratory, University of Burgundy, 12 rue de la Fonderie, 71200 Le Creusot, France

**Keywords:** Intravenous catheterization, NIR imaging, Illuminants, Subcutaneous veins, Image quality

## Abstract

**Background:**

Subcutaneous veins localization is usually performed manually by medical staff to find suitable vein to insert catheter for medication delivery or blood sample function. The rule of thumb is to find large and straight enough vein for the medication to flow inside of the selected blood vessel without any obstruction. The problem of peripheral difficult venous access arises when patient’s veins are not visible due to any reason like dark skin tone, presence of hair, high body fat or dehydrated condition, etc.

**Methods:**

To enhance the visibility of veins, near infrared imaging systems is used to assist medical staff in veins localization process. Optimum illumination is crucial to obtain a better image contrast and quality, taking into consideration the limited power and space on portable imaging systems. In this work a hyperspectral image quality assessment is done to get the optimum range of illumination for venous imaging system. A database of hyperspectral images from 80 subjects has been created and subjects were divided in to four different classes on the basis of their skin tone. In this paper the results of hyper spectral image analyses are presented in function of the skin tone of patients. For each patient, four mean images were constructed by taking mean with a spectral span of 50 nm within near infrared range, i.e. 750–950 nm. Statistical quality measures were used to analyse these images.

**Conclusion:**

It is concluded that the wavelength range of 800 to 850 nm serve as the optimum illumination range to get best near infrared venous image quality for each type of skin tone.

## Introduction

Hyperspectral and multi spectral imaging are well established systems used for remote sensing, satellites imaging, agriculture, physics and military applications. Recent years, these systems grasped attention of researchers in the field of biomedical imaging, especially where the standard imaging techniques fail to provide the desired outcomes [1]. Hyperspectral sensor captures the spectral information of an entire field of view for each band (or wavelength) storing the collected information as a data cube which contains spatial information for each wavelength or band, depending on the characteristics of the system used [2]. Hence, high spectral resolution will provide the ability to analyze data on wavelength or sub-wavelength scale. Furthermore, it also provides the ability to see beyond the visible range in order to acquire more information about the scene. For the case of skin and veins detection, hyper spectral venous imaging allows to look deeper below the skin in near infrared (NIR) range to find the best wavelength range for better contrast between skin and veins. This paper presents the analysis of hyperspectral data for the pre-selection of wavelength ranges suitable for the illumination in the process of veins localization.

Intravenous (IV) catheterization is the first and one of the most important steps in majority of medical treatments. Studies have reported that around 80% of all hospitalized patients and several outpatients need IV catheterization for blood sampling, medication delivery, transfusion and infusion of fluids to the patients [3,4]. The localization of veins is a tedious process which has to be done by medical practitioners prior to IV catheterization. Veins are usually localized by sight and/or feelings by pressing (with fingers) the targeted area of patient’s body, mostly forearm and hand. These methods of veins localization are approximate, depends on practitioner skills and often fail for the patients whose veins are neither easily visible nor be localized with feelings. The probability that a patient have veins non-visibility problem is high for people with dark skin tone, young children (especially infants), obese and dehydrated patients. The veins non-visibility problem leads to multiple attempts of catheter insertion which cause unnecessary pain and trauma to the patients. Studies have reported that failure to first attempt in general population is around 12 to 26% and it is worse in children 24 to 54% [5-7].

There are many devices available to help medical staff to better visualize the non-visible veins of patients. These devices use one of these techniques: Trans-illumination, Photo-acoustic, Ultrasound and NIR imaging. All these techniques have different advantages and drawbacks but NIR imaging is considered the most suitable for veins localization in catheterization process [8,9]. Indeed, NIR imaging uses non-ionizing light rays to penetrate deep inside skin tissues to acquire the image of venous structure. In electromagnetic spectrum, there is a low absorption window within NIR region, in which light can penetrate deeper inside skin tissues. This is due to the low absorption spectra of hemoglobin, oxy-hemoglobin and water which are main absorbers of radiations in skin. The span of this low absorption window is from 750 to 950 nm within NIR region [10]. Different NIR imaging devices use different wavelength or range of wavelength out of this low absorption window to illuminate the target site [11-14]. In [8] a NIR imaging method for subcutaneous veins localization is reported. In this work the illumination range used was spanning the full NIR low absorption window i.e. 740–950 nm (7 wavelengths with 8 LEDs for each). This lighting system can be used on a bench top system which has no power and space limitation but not suitable for a battery powered portable systems. In [9] no prior classification of skin is done with respect to skin tones. A monochrome camera with CMOS sensor was used to acquire images. For illumination 6 different wavelengths (740,770,810,840 and 910 nm) were used in 63 different combinations. No conclusion on final selection of illumination range was made.

This paper discusses the outcome of the analysis done on hyperspectral images for optimum illumination selection for the four classes of skin tones. A dataset of 80 subjects is made by scanning the forearm of each subject with a hyperspectral camera setup (detailed in next section). As the analysis studies the skin tone, the subjects are separated equally into four different classes based on the luminance measurement of their skin, obtain from a chromameter. To define optimum ranges suitable for illumination, NIR window is divided into four sub-bands (50 nm each) and mean images are formed for each sub-band. These images are analyzed using mean square error (MSE) and universal image quality index (Q) to define the best wavelength ranges where the image quality is highest when compared to the mean reference image.

## Hyperspectral image acquisition setup

**Figure 1:**
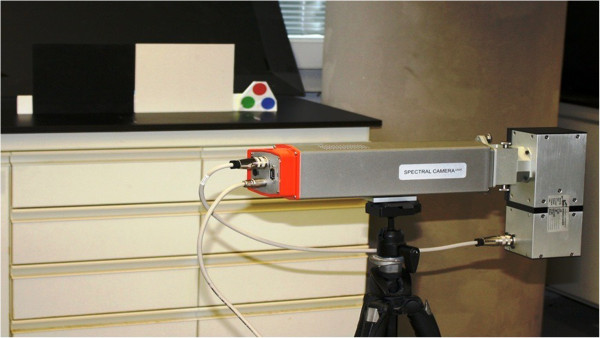
Hyperspectral image acquisition setup.

**Figure 2:**
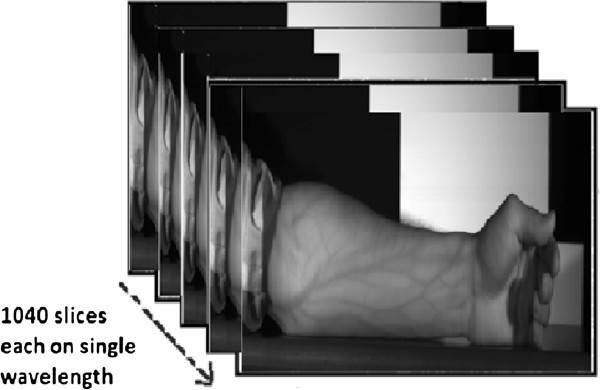
A hyperspectral cube, acquired from the hyperspectral image acquisition setup.

Hyperspectral imaging approach has been adopted to get deeper understanding of the effect of illuminants on the skin tissues and veins contrast. The idea was to acquire and to process hyperspectral images in visible and NIR range. The hyperspectral image acquisition system is shown in the Figure 1. Specim® Spectral Camera PS V10E has been used; it has the ability to acquire images in the visible as well as the NIR range i.e. 380 to 1055 nm with a spectral resolution of 2.8 nm which is the width of each band of captured spectrum. Total number of bands are 1040, which give an average distance of 0.65 nm between each central wavelength. This camera provides a high spatial and spectral resolution with adjustable field of view to be scanned. We acquired the images of forearm area of each subject, as the subcutaneous veins of this region are of our interest. Projector lamps (halogen) with a constant illumination range from 350 to 2500 nm were used to illuminate the targeted region. Each acquired hyperspectral image is in the form of cube with the spatial resolution of 450 × 1310 and 1040 spectral bands. A complete image of the scene on every single wavelength is acquired and saved in the form of cube (see Figure 2). This hyperspectral approach allows us to look deeper into the spectral response of skin tissues and veins against the wavelength of illumination.

Spectral DAQ software from Specim® was used to set the camera parameters and data acquisition. The angle for mirror scanner was set in the way that it should scan only the forearm region of the subject, allowing minimizing the data size of each hyper-spectral cube for each patient. The scanning time of hyperspectral image for one subject is about 40s. It depends on the selected scanning range of mirror scanner used with Specim VNIR Image sensor. Our targeted area was the fore arm region of subject, hence around 21° of scanning range (which covers the region from elbow to the tip of subjects fingers) is selected. The distance of mirror scanner is set to be about one meter from the subject’s position. The spectral images acquired are in the form of data cubes which are saved in environment for visualizing images (ENVI) compatible formats to be further processed. MATLAB is used to read the raw data cubes using ENVI toolbox.

A total of 80 volunteers were recruited for the data acquisition process. Most of these volunteers were students having body mass index (BMI) in healthy range .On the basis of BMI, 51 subjects have classified in healthy range, 7 in underweight, 15 in overweight and 7 in obese category [15].

**Figure 3:**
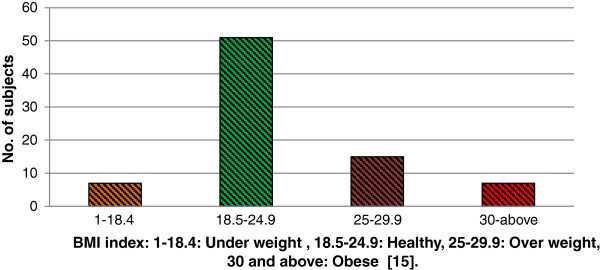
BMI histogram plot of the 80 subjects in dataset.

Patients having higher BMI are more likely to have non-visible veins; this is due to deeper veins due to higher proportion of fat in the hypodermis layer of skin [6,16]. The plot of BMI spread is given in Figure 3.

## Skin tone classification

The skin tone is an important factor which affects the veins localization process. People with darker skin are more likely to be affected with a non-visibility of their veins . In order to achieve better results of veins viewing in subjects having different skin tones, we classified the skin into four classes. These classes are: Fair, Light-brown, Dark-brown and Dark. CIE L*a*b values for each subject were obtained with the help of chromameter (Konica Minolta Inc.,). Data from three different locations for each forearm per subject were acquired so as to infer a mean value. The chromometer is placed on wrist, central and near elbow region of each subject to get three readings. An average value of luminance (L*) for each subject is then fed into a Fuzzy C-Mean (FCM) algorithm. FCM is a clustering technique which allows the data to belong to two or more clusters at the same time. Based on the membership degree, decision of the parent cluster is made for each data point [17]. As a result, skin tone of each subject has been classified into one of the four classes. The FCM objective function is given by Eq.1 as follows:

(1) $$J_{m}=\sum_{i=1}^{N}\sum_{j=1}^{C}u_{\mathrm{ij}}^{m}{ǁ}_{xi}\;-\;c_{j}{ǁ}^{2},\;1\;\leq \;m\;<\;\infty$$

**Figure 4:**
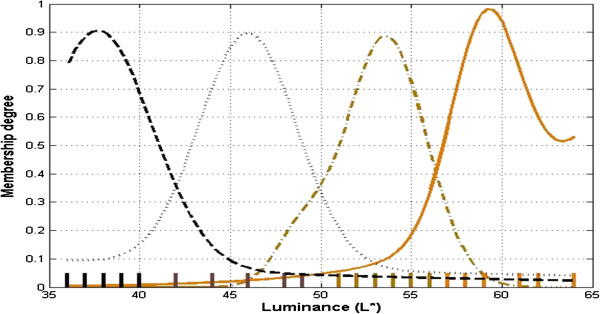
Skin tone classification using FCM in to four classes: fair, light brown, dark brown and dark.

**Figure 5:**
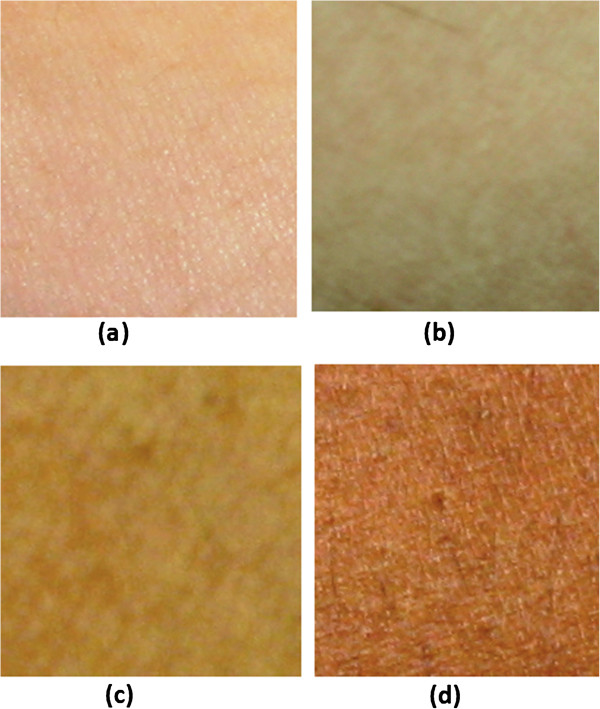
**Digital (zoomed) image of four skin classes: a) fair, b) light brown, c) dark brown and d) dark skin.** Images acquired with Canon EOS 500D indoor fluorescent lighting.

Here N is the number of data points, C is the number of clusters, m is any real number having value greater than 1, xi is the ith data point, uij is the degree of membership of current data point in the cluster J and cj is the centroid of cluster j.Figure 4 depicts the classification of skin tone in four different classes using FCM classifier. The membership function is constructed by applying Gaussian fitting to the data set. The small bar lines on x-axis show the occurrences of the L* value in data set. Note at same point on x-axis multiple occurrences can be possible but those are shown by single bar only. Figure 5 shows the digital colour image (zoomed) of skins that belongs to the four different classes (i.e. fair, light brown, dark brown and dark).

## Quality analysis of mean images

A mean NIR image (Im) was created for each subject, by taking mean of bands over a spectral range from 750 - 950 nm. This spans the whole NIR low absorption window. The low absorption window is the range of wavelength from 750 to 950 nm in which main absorbers of skin have low absorption coefficient, which allows the radiations (NIR light) to penetrate deeper in the skin tissues [10]. These mean NIR images serve as reference images since the contrast per pixel (cpp) for these images is higher than the mean images obtained in other spectral regions. Moreover, the noise, present in each band (of 2 nm) is reduced in the mean image due to its highly random nature. The idea is to find the optimum illumination range within NIR region. There are no hard boundaries between bands and the spectrum seems continuous. Four sub-mean images are created by taking mean of bands in the following ranges:

Im: mean 750-950 nm

Image-1: mean 750-800 nm

Image-2: mean 800-850 nm

Image-3: mean 850-900 nm

**Figure 6:**
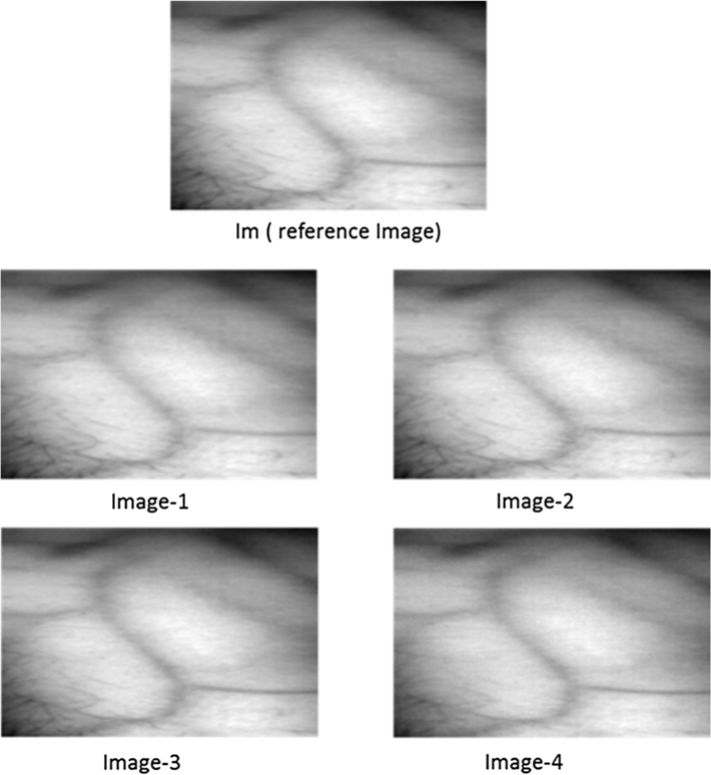
**I**_**m **_**(reference image) and four sub-mean images for fair skin subject.**

**Figure 7:**
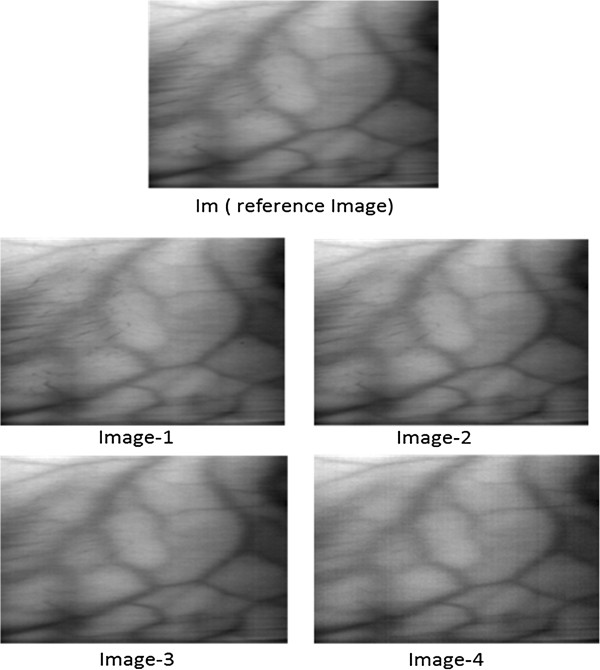
**I**_**m **_**(reference image) and four sub-mean images for light brown skin subject.**

**Figure 8:**
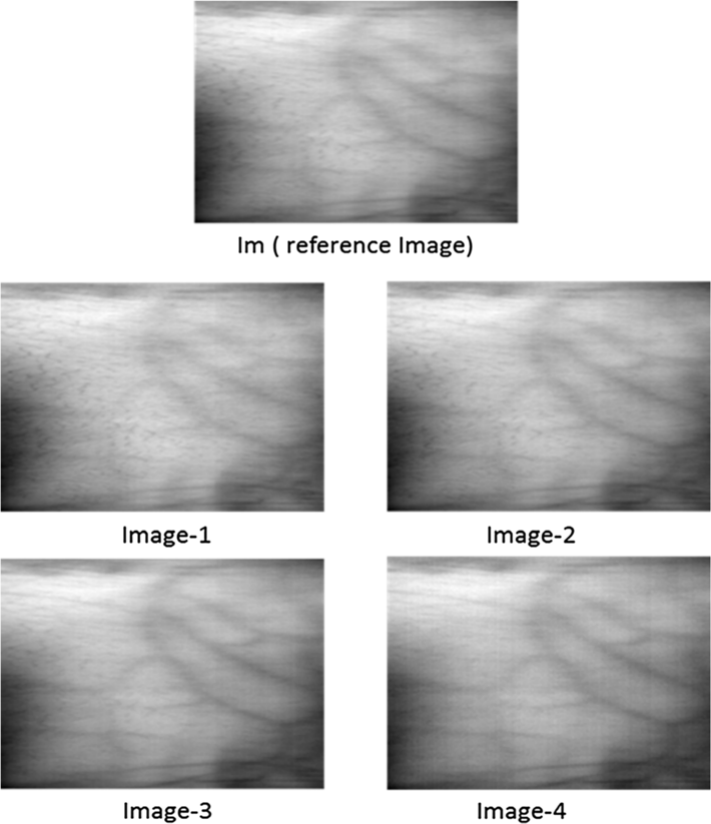
**I**_**m **_**(reference image) and four sub-mean images for Dark Brown skin subject.**

**Figure 9:**
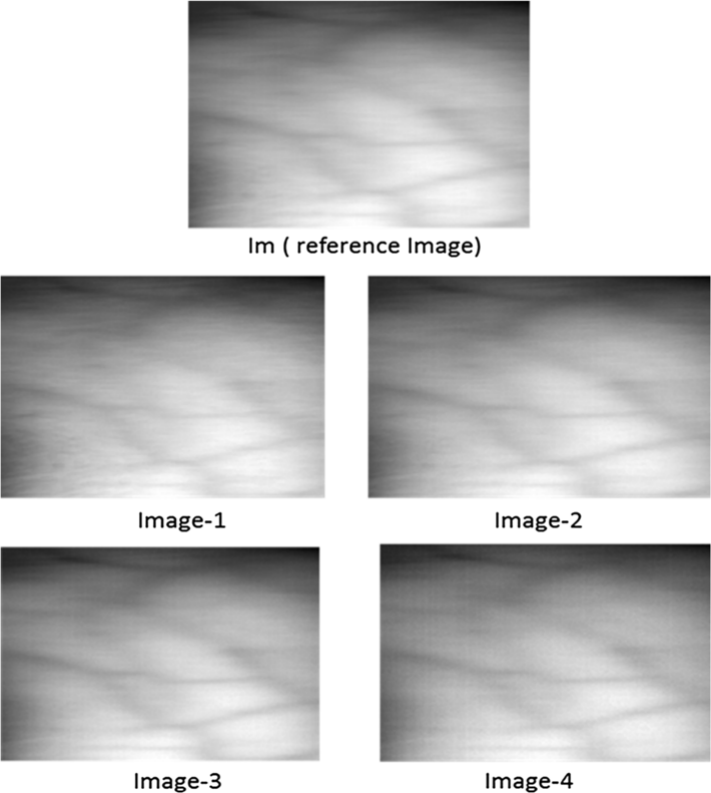
**I**_**m **_**(reference image) and four sub-mean images for dark skin subject.**

Image-4: mean 900-950 nmFrom the hyper spectral dataset, 12 subjects from each skin class were selected randomly. With the data from selected subjects, mean reference image called Im and four sub-mean images, named Image1-4, were created for each subject of all four classes. Figure 6, Figure 7, Figure 8 and Figure 9 depicts reference images Im and four sub-mean images for a random subject among each skin class. These sub-mean images were then analyzed with the reference of mean reference image Im.

The ultimate users of images are human beings. The most trustworthy way of quality assessment of images is subjective analysis. It is based on human visual system (HVS). The mean opinion score (MOS) is measured by the human viewers. However, this measure is expensive and time consuming [18]. Furthermore, the MOS is severely affected by the image viewing conditions. In this work objective image quality assessment is chosen due to the following reasons.

1. Human subjects are unable to distinguish between image quality of all sub-mean images since they look quite similar.

2. Contrast between veins and skin tissues cannot be determined fairly based on the human visual system.

3. The acquired NIR images are fed to veins detection algorithm which provide an objective assessment observation.

Objective quality measurement is important for machine vision applications. Mathematical measures are used to measure and compare the image quality w.r.t. reference images. The factors, like mean square error (MSE) and Universal Image Quality Index (Q) are widely used to calculate the quality of images with a reference image which is considered the best image of the scene [19]. In this work, these two factors are chosen to find out the best range of wavelengths on which one can have good quality image.

Both images (Im and sub-mean images) are converted in to 1-D vector before calculating the MSE. For simplicity of notation, we named reference image Im as 'x’ and the sub mean image for which we want to calculate MSE and Q as 'y’.

(2) $$\mathrm{MSE}=\frac{1}{N}\sum_{i=1}^{N}{\left(\;x_{i}\;-\;y_{i}\right)}^{2}$$

Where 'N’ is the total number of pixels in both images. Furthermore ’xi’ and 'yi’ is the ith pixel in image x and y respectively [20]. Image x is the reference image and Image y is the one for which we want to calculate the MSE value. The universal image quality index is a measure that is independent of viewing conditions. The range of Q is [-1, 1] and is defined by the following equation:

(3) $$Q=\frac{\sigma_{\mathrm{xy}}}{\sigma_{x}\sigma_{y\;}}\;\times \frac{2\bar{x}\;\bar{y}}{{\left(\bar{x}\right)}^{2}+{\left(\bar{y}\right)}^{2}}\;\times \frac{2\sigma_{x}\sigma_{y\;}}{\sigma_{x}^{2}+\sigma_{y\;}^{2}}$$

*Where*$\bar{x}=\frac{1}{N-1}\sum_{i=1}^{N}x_{i}\cdot \cdot \cdot \cdot \cdot \cdot \cdot \cdot \cdot \cdot \cdot \cdot \cdot \cdot \cdot \cdot \cdot \bar{y}=\frac{1}{N-1}\sum_{i=1}^{N}y_{i}$

$$\sigma_{x}^{2}=\frac{1}{N-1}\sum_{i=1}^{N}{\left(\;x_{i}\;-\;\bar{x}\right)}^{2},\;\sigma_{y}^{2}=\frac{1}{N-1}\sum_{i=1}^{N}{\left(\;y_{i}\;-\;\bar{y}\right)}^{2}$$

$$\sigma_{x}\sigma_{y}\;=\frac{1}{N\;-\;1}\sum_{i=1}^{N}\left(x_{i}\;-\;\bar{x}\right)\left(y_{i}\;-\;\bar{y}\right)$$

**Figure 10:**
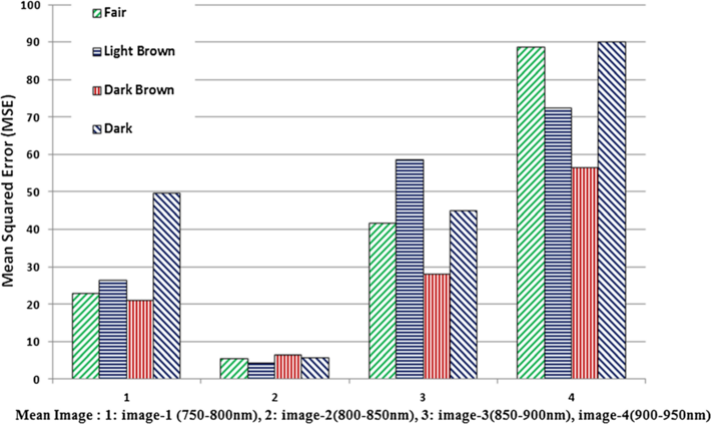
MSE plot for four sub-mean images of four skin classes.

**Figure 11:**
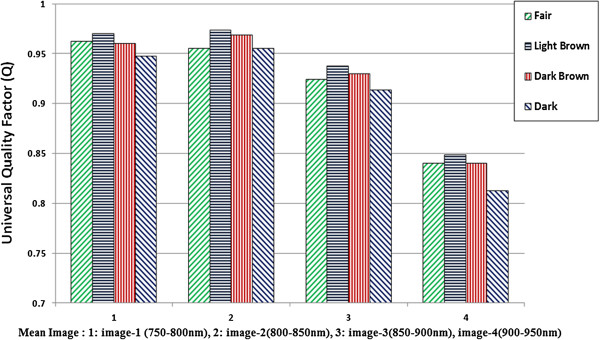
Universal quality factor (Q) plot for four sub-mean images of four skin classes.

There are three components of Q in Eq.3; the first component is the coefficient of correlation between images, the reference image and the one whose quality factor is being measured. With the second component, the relation of luminance of both images is measured. Third component measures the similarity of contrast of both images.In Figure 10 the mean value of MSE calculated for four sub-mean images for all 12 subjects of each class is plotted. In this plot it can be observed that the MSE for the sub-mean image (Image-2), which was formed by taking mean in the range of 800 -850 nm bands, has the lowest value of MSE for all skin classes. The 4th image (Image-4) which was formed by taking mean in the range of 900- 950 nm bands has the highest MSE for all skin classes.The Q factor is plotted in Figure 11. In this plot the value of Q factor is higher for Image-2 as compared to other 3 images except in case of fair skin. In that case the Q value for Image-1 is slightly higher than Image-2, but the difference is not that big which can lead to any conclusive remarks. The overall results are consistent with the MSE values.

Through this work, it is determined that the image (image-2) which was made with the mean of bands in range 800-850 nm from multispectral data has the best quality. The measure like MSE, PSNR and universal image quality (Q) is found best for this range. Keeping in mind the image acquisition setup, it is concluded that the best quality image is obtained in the spectral range of 800 – 850 nm. These findings will serve the basis of optimum illumination selection for a NIR system for subcutaneous veins localization. For this system, our choice will be the LEDs with a central wavelength lying within 800-850 nm range. With this optimized illumination it is anticipated that better quality and high contrast NIR images can be obtained.

## Conclusion

Near infrared imaging is proven as a best technique in veins visualization systems. Optimized illumination is crucial for the low power, portable and wearable systems. A hyperspectral analysis has been done in this work to get the best range of illumination wavelength, for the better image quality and higher contrast. The NIR region is divided into 4 sub regions and means images were formed from these sub-regions. These mean images are analysed with objective quality measurement factors like MSE and Universal Image quality Factor 'Q’ to define best range for illumination. It has been found that the range 800 to 850 nm provides best image quality. For Fair skin, the image quality is slightly better in the range of 750 to 800 nm. For darker skin the overall range considered suitable is 800 to 850 nm. This infers that if we choose the illumination within this range, we can have better image quality and higher contrast for venous images.

Future work is to make the prototype system with optimized illumination and compare the results with a system with generalized illumination.

## Competing interests

The authors declare that they have no competing interests.

## Authors’ contributions

AS, MNS and NW conceived the study and participated in its design and coordination and helped to draft the manuscript. AS and NW performed the data acquisition and analysis in the Universiti Teknologi PETRONAS. ASM and FM provided guidance in every part of this work and assisted in the writing and editing of the manuscript. All authors read and approved the final manuscript.
